# High-risk biochemical recurrence of locally treated prostate cancer after EMBARK. An end to decades of conventional wisdom?

**DOI:** 10.1038/s41391-023-00767-y

**Published:** 2023-12-08

**Authors:** Vérane Achard, Bertrand Tombal

**Affiliations:** 1 Radiation Oncology, HFR Fribourg, Villars-sur-Glâne, Switzerland; 2 Faculty of Medicine, University of Geneva, Geneva, Switzerland; 3 Institut de recherche clinique, Université catholique de Louvain, Brussels, Belgium

**Keywords:** Cancer therapy, Cancer screening

Managing biochemical recurrence (BCR) of localized prostate cancers (PCa) that have failed all options for local treatment has been the topic of an incommensurable number of manuscripts and opinion papers.

Freedland et al. reported the results of EMBARK in the October edition of the *New England Journal of Medicine* [1]. EMBARK has randomized 1068 patients with a high-risk BCR to androgen deprivation therapy (ADT) alone, ADT + enzalutamide, or enzalutamide alone. At five years, the metastasis-free survival (MFS) was 87.3% with ADT + enzalutamide, 71.4% with ADT alone, and 80.0% with enzalutamide monotherapy. ADT + enzalutamide delays MFS over leuprolide (hazard ratio (HR) 0.42; *p* < 0.001), which is not a surprise. Noticeably, enzalutamide monotherapy also delays MFS over ADT alone (HR 0.63; *p* = 0.005). The patient-reported outcome measures show that enzalutamide combination and monotherapy vs. ADT preserve high health-related quality of life (HR-QoL) [2]. The least we can say is that these results will change practice in many respects.

The BCR population is heterogeneous. Many patients won’t develop metastases. Efforts have been made to stratify that population. The disease trajectory can be estimated by combining the Gleason score, the interval between the local treatment and the BCR, and, more importantly, the PSA doubling time (PSADT). In Freedland’s landmark publication, the HR for the time to PCa-specific death after BCR following radical prostatectomy was 3.53 (*p* = 0.002) for interval ≤ 3 vs. > 3 years, 2.26 (*p* = 0.002) for a pathological Gleason score ≥ 8 vs. < 8, and 8.76 (*p* < 0.0013) for a PSADT between 3.0 and 8.9 vs. ≥ 15 months [3]. Similar observations have been made after radiotherapy. There have been many attempts to define an optimal definition of high/low-risk BCR integrating PSA kinetic and pathological characteristics, hence leading to several definitions with different prognostic value field [4]. The EAU guidelines stratify BCR patients into low and high risk, the latter being the patients with PSADT < 1 year or a pathological ISUP grade 4–5 after radical prostatectomy and an interval to biochemical failure < 18 months or a biopsy ISUP grade 4–5 for after radiotherapy [5].

The only treatment to decide on for many years was ADT. No study has definitively shown that immediately starting ADT improves OS. The largest trial so far randomized 293 men between immediate and delayed ADT [6]. Interestingly, the definition of delayed included a decrease in PSADT ≤ 6 months. Five-year OS were 86.4% and 91.2% in the delayed and immediate ADT arms, respectively (HR 0·55, *p* = 0·050). ADT also has side effects, including hot flashes, loss of libido, and cognitive decline [5]. Administered over a long period, ADT induces metabolic changes that can lead to an increased risk of cardiovascular events and decreases bone mineral density, leading to an excess risk of fracture. Consequently, physicians usually consider that the risk of ADT outweighs its benefits and generally agree that strategies aiming at delaying ADT are acceptable. The guidelines are more prudent, stating that immediate ADT should not be offered to low-risk patients.

In that context, physicians have enthusiastically embraced new imaging techniques and metastatic-directed therapies (MDT) as an alternative to immediate ADT. Many studies demonstrate that PET/CT with diverse radioligands significantly outperforms bone and CT scans [7]. Applied in the setting of BCR, they will robustly identify metastatic deposits earlier, usually at the stage of few metastases [8]. MDT was raised as a standard treatment because these metastases were technically amenable to targeted treatment, usually stereotactic radiotherapy. However, the evidence needs to be stronger. The concept is supported only by two phase II trials with 116 patients with low and high-risk BCR [9]. These trials do not demonstrate that MDT increases MFS, prolongs OS, or improves HR-QoL. They suggest that ADT can be postponed. New imaging technologies and MDT have thus occupied the field of BCR treatment on “conventional wisdom”, a concept described by the economist JK. Galbraith as “the ideas that are esteemed at any time for their acceptability” in his 1958 book “*The Affluent Society*”.

But is it still acceptable to delay systemic treatment now that we have potent AR pathway inhibitors (ARpI) that significantly delay MFS in patients with high-risk BCR? In contrast to delaying ADT, MFS is a surrogate for OS in recurrent prostate cancer [10]. Delaying ADT may have been acceptable, but delaying enzalutamide for high-risk BCR is not acceptable anymore unless we have strong evidence. EMBARK establishes ARpI as the new standard of care for patients with a high-risk BCR. A rapid rise in PSA, even more in the context of an ISUP 4–5 cancer or a short interval to BCR, may be seen as a **trigger to treat** and no longer a trigger to scan to decide whether a treatment is needed or not. Whether a patient has metastases or not, a few or a lot, he needs enzalutamide right away. Because the standard of care has changed, new questions will emerge, involving new imaging technologies.

What is the role of ADT when systemic treatment is needed? EMBARK demonstrates that enzalutamide alone offsets ADT alone in terms of MFS. So, the question today is not when an ARpI should be added to ADT but when ADT should be added to the ARpI. The answer will be complex. In EMBARK, adding ADT to enzalutamide has an incremental benefit in high-risk BCR patients [1]. As for the quality of life, the results published so far used instruments lacking the granularity to correctly identify the potential benefit of ARpI monotherapy [2].

As for the use of new imaging technologies and MDT, they should leave the front scene but may gain interest in serving other causes. EMBARK incorporates intermittent treatment for patients with PSA decreases to < 0.2 ng/ml at week 37, complying with the standard of care established by the NCIC PR-7 trial [11]. In EMBARK, the treatment’s pause ranges from 11 months with enzalutamide to 20.2 months with enzalutamide and ADT. Extending that “off-period” has become a new target for future trials. This creates a new opportunity for MDT and a broader approach, including radioligand therapies.

EMBARK disrupts decades of beliefs that ADT monotherapy is the backbone of systemic treatment for prostate cancer. Starting from now, the backbone is an ARpI and future trials should be built on that.
